# Telomere length, pre-eclampsia, and gestational diabetes

**DOI:** 10.1186/1756-0500-3-113

**Published:** 2010-04-23

**Authors:** Emily W Harville, Michelle A Williams, Chun-fang Qiu, Julie Mejia, Rosa Ana Risques

**Affiliations:** 1 Department of Epidemiology, Tulane School of Public Health and Tropical Medicine, New Orleans, LA, USA; 2 Center for Perinatal Studies, Swedish Medical Center, Seattle, WA, USA; 3 Department of Epidemiology, University of Washington School of Public Health and Community Medicine, Seattle, WA, USA; 4 Department of Pathology, University of Washington, Seattle, WA, USA

## Abstract

**Background:**

Telomere length is a marker of cumulative damage to the cell, and has been associated with cardiovascular disease, hypertension, and diabetes.

**Findings:**

The association of telomere length with pre-eclampsia and gestational diabetes mellitus (GDM) was examined in a nested case-control study. Circulating leukocyte telomere length was measured by Quantitative-PCR. Mean and median telomere length among cases and controls was compared, and logistic regression was used to model the outcomes as a function of tertile telomere length, with control for effects of potential confounders. Mean telomere length in pre-eclampsia cases was 0.77 (SD 0.14), in GDM cases was 0.73 (SD 0.10), and in controls was 0.74 (SD 0.14). The adjusted odds ratio comparing the highest tertile to the lowest for pre-eclampsia was 0.92 (0.15-5.46), and for gestational diabetes was 0.65 (0.13-3.34).

**Conclusions:**

Further study is necessary to determine if telomere length is associated with these pregnancy complications.

## Background

Telomeres are terminal regions of chromosomes that comprise multiple tandem repeats of a base sequence[1] Telomere length is a marker of the cumulative damage the cell has been exposed to [2], and has been associated with cardiovascular disease[3], general cardiovascular damage [4], and atherosclerosis [5]. Hypertensive patients have been reported to have shorter telomeres [6-9], especially when they had high renin to aldosterone ratios[10]. Similarly, blood pressure has been associated with shorter telomeres and low telomerase activity [11-13] Diabetes patients have been shown to have shorter telomeres in several studies, [11,14-17] and increased telomere attrition [18] and shorter telomere length [6,19] have been associated with insulin resistance. Higher glucose and insulin were associated with shorter telomeres [11,20] and low telomerase activity [12], while good glycemic control was associated with more favorable telomere dynamics[17,20]

Telomere length and dynamics vary substantially between individuals [21] and are influenced by a number of factors, including genetics and family relationship [13,22,23], multivitamin use [23], race [21], smoking [12,21,24], and body mass index [6,18,24].

Pre-eclampsia is associated with increased risk of cardiovascular disease [25] and gestational diabetes is associated with increased risk of later diabetes[26], suggesting that risk factors and etiologies of cardiovascular disease and diabetes and these outcomes is likely to be similar. Thus, a reasonable hypothesis is that telomere length will also differ in patients with these outcomes. In addition, telomere length has been associated with oxidative stress, [16,27] and markers of oxidative stress are raised in pre-eclampsia[28] A recent article found lower telomere length in the placentas of women with pre-eclampsia and intrauterine growth retardation[29] We are unaware of other previous studies of telomere length in pre-eclampsia and gestational diabetes. For studies related to reproductive outcomes, no difference in telomere lengths was found in young adults whose mothers had pre-gestational type 1 diabetes[30], while another study of newborns did not find statistically significant differences in telomere lengths between newborns whose mothers had pre-eclampsia, hypertension, or diabetes[31]. However, these studies dealt with the child rather than the mothers.

Recurrent miscarriage has also been associated with shorter telomere length [32], but no difference in telomere length by small-for-gestational-age was seen[22] We examined the relationship between pre-eclampsia, gestational diabetes, and telomere length in a nested case-control study.

## Methods

50 cases of pre-eclampsia, 25 cases of GDM, and 50 controls were selected from an existing study, conducted from April 1998 to June 2002 in Washington State. This study has been described in detail elsewhere [33]. Using the then-current guidelines, preeclampsia was defined as sustained pregnancy-induced hypertension with proteinuria. Hypertension was defined as sustained blood pressure readings of ≥ 140/90 mmHg (with readings taking place ≥ 6 hours apart) and/or a sustained 15 mm Hg diastolic rise or a 30 mm Hg systolic blood pressure above first-trimester values. Proteinuria was defined as urine protein concentrations of ≥ 30 mg/dl on ≥ 2 random specimens collected at least 4 hours apart. In our study setting, according to the recommendations from the American Diabetes Association (ADA) [34] pregnant women were screened at 24-28 weeks gestation using a 50 gram 1-hour oral glucose challenge test. Those patients who failed this screening test (glucose ≥ 7.8 mmol/L) were then followed-up within 1-2 weeks with a 100 g, 3-h oral glucose tolerance test (OGTT). We also abstracted laboratory results from participants' 50 gram 1-hour glucose challenge test and from the diagnostic 100 gram 3-hour OGTT. Women were diagnosed with GDM if two or more of the 100 gram OGTT glucose levels exceeded the ADA criteria [34]: fasting >5.3 mmol/L; 1-hour >10.0 mmol/L; 2-hour >8.6 mmol/L; 3-hour >7.8 mmol/L.

Controls were women who delivered within two hours of a case with pregnancies uncomplicated by pregnancy-induced hypertension. A structured interview questionnaire, administered during participants' postpartum hospital stay, was used to collect information on maternal sociodemographic, medical, reproductive, and lifestyle characteristics during in-person interviews. The women chosen for this analysis were required to be between 20-35 years old, non-Hispanic white, with no pre-existing hypertension or diabetes, and carrying a singleton pregnancy.

Non-fasting blood samples were collected in 10 ml tripotassium EDTA Vacutainer tubes during the intrapartum period. These were protected from ultraviolet light, kept on wet ice, and processed within 30 minutes of phlebotomy. Plasma was decanted into cryovials and kept frozen at -70°C or below until analysis.

Leucocyte telomere length (hereafter referred to as telomere length) was measured by Quantitative-PCR using the method described by Cawthon [35]. Briefly, for each sample, two PCRs are performed: the first one to amplify the telomeric DNA and the second one to amplify a single-copy control gene (36B4, acidic ribosomal phosphoprotein PO). This provides an internal control to normalize the starting amount of DNA. The amount of telomeric DNA is divided by the amount of control-gene DNA, producing a relative measurement of the telomere length of the sample. The average inter-experimental coefficient of variation was 0.05.

Sample size for this study was determined based on other studies of telomere length. Comparing groups of 50 gives us better than 90% power to see a difference as large as that seen in a paper demonstrating reduced telomere length under chronically stressful conditions [36]. Fifty per group gives us 80% power to see an odds ratio of 3.2 for top vs. bottom quartile, lower than the hazard ratio of 3.5 seen in previous studies of telomere length and cancer [37]. Several studies of this size looking at telomere length and reproductive outcomes have been published[22,30,32]

Mean and median telomere length among cases and controls was compared using t-tests and Wilcoxon nonparametric tests. Telomere length was divided into tertile based on the control group, and logistic regression was used to model the outcomes as a function of tertile telomere length, with control for effects of potential confounders. In addition, the association between telomere length and other variables was examined within cases and controls.

Ethics approval for the initial study was granted by the University of Washington Institutional Review Board, and informed consent (including for sample banking) was gathered from all subjects. Tulane University IRB granted approval for this secondary analysis.

## Results

Pre-eclampsia cases were on average younger, heavier, more likely to smoke, less likely to have higher education, and more likely to have a family history of hypertension than controls (table 1). GDM cases were on average older, heavier, and more likely to have a family history of diabetes (table 2). Mean telomere length in pre-eclampsia cases was 0.77 (SD 0.14), in GDM cases was 0.73 (SD 0.10), and in controls was 0.77 (SD 0.14). If the pre-eclampsia cases were limited to those who gave birth preterm (n = 30), mean telomere length was 0.79 (SD 0.15), which was not significantly different from that of controls. Odds of pre-eclampsia were lowest in the middle tertile of telomere length and essentially equal in the lowest and highest tertiles in the unadjusted and both adjusted models (table 3). There was evidence of reduced risk of GDM with the highest tertile of telomere length, though the associations did not reach statistical significance in any of the 3 models tested.

**Table 1:** Distribution of Preeclampsia (PE) Cases and Normotensive Control Subjects According to Selected Characteristics, Seattle and Tacoma, Washington, 1998 - 2002.

	**PE Cases**		**Control Subjects**		**p**
			
	**(N = 50)**		**(N = 50)**		
	**n**	**%**	**n**	**%**	
Maternal Age (years)					<0.01
20-24	19	38	5	10	
25-29	13	26	14	28	
30-34	18	36	31	62	
Maternal Age (years)^†^	27.3 ± 0.6		29.5 ± 0.5		<0.01
Maternal Race/Ethnicity					
Non-Hispanic White	50	100	50	100	..
Unmarried	13	26	8	16	0.22
≤ 12 years Education	15	30	3	6	<0.01
Nulliparous	50	100	50	100	...
Smoked During Pregnancy	11	22	7	14.8	0.06
Pre-pregnancy BMI*^†^	26.9 ± 0.9		22.4 ± 0.4		<0.01
Pre-pregnancy BMI*					<0.01
< 20	1	2	13	26	
20-24.9	25	50	27	54	
25-29.9	11	22	8	16	
≥ 30	13	26	2	4	
Annual Household Income (US$)					0.36
<30,000	9	18	9	18	
30,000-69,999	24	48	17	34	
70,000+	17	34	23	46	
Unknown	0	0	1	2	
Physical inactive during pregnancy	24	48	16	32	0.10
Family history of chronic hypertension	29	58	17	34	0.02
Gestational age at delivery (weeks)	34.4 ± 0.6		39.7 ± 0.2		<0.01
Time from last meal to blood drawn (hours)	3.03 ± 0.53		1.68 ± 0.19		0.02
Telomere length^†^	0.77 ± 0.02		0.77 ± 0.02		0.75
Median (inter-quartile range)	0.75 (0.66, 0.83)		0.77 (0.70, 0.84)		0.56

*Pre-pregnancy body mass index = BMI = weight (kg)/height (m^2^).

^†^Mean ± SEM;

**Table 2:** Distribution of Gestational Diabetes Mellitus (GDM) Cases and Normotensive/Euglycemic Control Subjects According to Selected Characteristics, Seattle and Tacoma, Washington, 1998 - 2002.

	**GDM Cases**		**Control Subjects**		**p**
			
	**(N = 25)**		**(N = 50)**		
	**n**	**%**	**n**	**%**	
Maternal Age (years)					0.29
20-24	3	12	5	10	
25-29	3	12	14	28	
30-34	19	76	31	62	
Maternal Age (years)^†^	30.9 ± 0.7		29.5 ± 0.5		0.08
Maternal Race/Ethnicity					
Non-Hispanic White	25	100	50	100	---
Unmarried	3	12	8	16	0.74
≤ 12 years Education	1	4	3	6	0.59
Nulliparous	12	48	50	100	<0.01
Smoked During Pregnancy	2	8	7	14.8	0.54
Pre-pregnancy BMI*^†^	27.8 ± 1.5		22.4 ± 0.4		<0.01
Pre-pregnancy BMI*					<0.01
< 20	3	12	13	26	
20-24.9	7	28	27	54	
25-29.9	7	28	8	16	
≥ 30	8	32	2	4	
Annual Household Income (US$)					
<30,000	5	20	9	18	0.93
30,000-69,999	7	28	17	34	
70,000+	12	48	23	46	
Unknown	1	4	1	2	
Physical inactive during pregnancy	10	40	16	32	0.49
Family history of diabetes mellitus	4	16	3	6	0.21
Gestational age at delivery (weeks)	39.0 ± 0.2		39.7 ± 0.2		0.05
Time from last meal to blood drawn (hours)	3.25 ± 0.74		1.68 ± 0.19		<0.01
Telomere length^†^	0.73 ± 0.02		0.77 ± 0.02		0.20
Median (inter-quartile range)	0.72 (0.69, 0.76)		0.77 (0.70, 0.84)		0.10

*Pre-pregnancy body mass index = BMI = weight (kg)/height (m^2^).

^†^Mean ± SEM;

**Table 3:** Odds Ratios (OR) and 95% Confidence Intervals (CI) for Preeclampsia (PE) and gestational diabetes mellitus (GDM) according to Tertiles of Maternal Blood DNA Telomere Length Sampled at Delivery, Seattle, Washington, 1998-2002.

Telomere length	Preeclampsia (N = 50)	Controls	Unadjusted OR	Age-adjusted OR	Adjusted OR*
		(N = 50)	(95%CI)	(95%CI)	(95%CI)
Tertile 1 (<0.720)	23	19	1.00 (reference)	1.00 (reference)	1.00 (reference)
Tertile 2 (0.720-0.800)	8	15	0.44 (0.15-1.26)	0.45 (0.15-1.35)	0.46 (0.62-3.38)
Tertile 3 (≥ 0.801)	19	16	0.98 (0.40-2.41)	1.08 (0.42-2.75)	0.92 (0.15-5.46)
					
P for trend			0.91	0.94	0.83
					
**Telomere length**	**GDM**	**Controls**	**Unadjusted OR**	**Age-Adjusted OR**	Adjusted OR*
	**(N = 25)**	**(N = 50)**	**(95%CI)**	**(95%CI)**	**(95%CI)**
					
Tertile 1 (<0.720)	14	19	1.00 (reference)	1.00 (reference)	1.00 (reference)
Tertile 2 (0.720-0.800)	6	15	0.54 (0.17-1.75)	0.56 (0.17-1.87)	0.96 (0.19-4.77)
Tertile 3 (≥ 0.801)	5	16	0.42 (0.13-1.43)	0.35 (0.10-1.23)	0.65 (0.13-3.34)
					
P for trend			0.15	0.09	0.61

The cutoffs of tertile were based on the distribution in controls

* adjusted for maternal age, pre-pregnancy body mass index, gestational age at delivery and time from last meal to blood draw.

Among controls, covariates were examined for relationships with telomere length. There was some evidence of association with telomere length for hours since last meal (r = 0.24, p = 0.09) and birthweight (r = 0.24, p = 0.10). Among the controls, mean telomere length was shorter in preterm infants (0.69 vs. 0.78, p = 0.26), ever smokers (0.74 and 0.79, p = 0.25), unmarried women (0.72 vs. 0.78, p = 0.18), and those who had eaten most recently (0.73 vs. 0.82, p = 0.05).

## Discussion

The main findings of the study are (1) no association of telomere length with pre-eclampsia, and (2) shorter telomeres associated with gestational diabetes, but differences might be due to chance. The findings for pre-eclampsia are in contrast with previous studies that have reported shorter telomeres in individuals with hypertension and blood pressure [6-9,11-13], as well as a study finding shorter telomeres in the placentas of pre-eclamptic women[29] It may be that telomere attrition in hypertension may take years to accumulate, at least in leucocytes, and thus is not apparent in acute pre-eclamptic hypertension. However, some prior studies that explored the association of telomere length with cardiovascular diseases also failed to find an association with hypertension, [11,38] suggesting that this association may depend on the characteristics of the study population.

We found a trend of shorter telomeres in women with gestational diabetes. This is in agreement with previous studies that have reported shorter telomeres in individuals with type 2 diabetes mellitus[11,14,16,19] The lack of statistical significance in our study is likely to be due to the small number of cases, as leukocyte telomere length is highly variable between individuals, or the trend we see could simply be due to chance variation. In addition, as with pre-eclampsia, variations in the pregnancy-related and chronic form of this disease and the characteristics of the study population could also account for the lack of significant association in this study.

The difference in mean telomere length for pre-eclampsia was 0.008, with a standard deviation of approximately 0.14. To have 80% power to see a difference that large, more than 5000 samples would need to be tested. To see an odds ratio of 0.92, approximately 8000 samples would be required. The difference in mean telomere length for GDM was 0.01, with a standard deviation of approximately 0.14. To have 80% power to see a difference that large, more than 3000 samples would need to be tested. To have 80% power to see an odds ratio of 0.65, approximately 325 cases and controls would be required. Thus, in both cases a much larger set of samples than that included in this study would be needed. Moreover, future studies should also address the analysis of additional markers of oxidative stress as well as leukocyte telomerase activity, as these parameters might help elucidate the biological and functional role of telomere shortening in these diseases.

## Conclusions

The odds ratio for GDM was lowest in the highest tertile of telomere length, but the associations did not reach statistical significance. No association was seen with pre-eclampsia. Further research is necessary to determine if telomere length is associated with pregnancy complications or outcomes.

## Abbreviations

GDM: gestational diabetes mellitus; ADA: American Diabetes Association; OGTT: oral glucose tolerance test.

## Competing interests

The authors declare that they have no competing interests.

## Authors' contributions

EH conceptualized the study and took the lead in writing the paper. MW supervised the pre-eclampsia and diabetes studies and assisted in writing the paper. CQ assisted in organizing the study and data analysis. JM and RR performed laboratory analyses and assisted in writing the paper. All authors read and approved the final manuscript.
